# MicroRNA-27a promotes renal tubulointerstitial fibrosis via suppressing PPARγ pathway in diabetic nephropathy

**DOI:** 10.18632/oncotarget.10283

**Published:** 2016-06-24

**Authors:** Xiaoyan Hou, Jianwei Tian, Jian Geng, Xiao Li, Xun Tang, Jun Zhang, Xiaoyan Bai

**Affiliations:** ^1^ Division of Nephrology, Nanfang Hospital, Southern Medical University, National Clinical Research Center for Kidney Disease, State Key Laboratory of Organ Failure Research, Guangdong Provincial Institute of Nephrology, Guangzhou, Guangdong, PR China; ^2^ Department of Nephrology, Zhujiang Hospital, Southern Medical University, Guangzhou, Guangdong, PR China; ^3^ Department of Nephrology, The First Affiliated Hospital, Inner Mongolia Medical University, Hohhot, Inner Mongolia, PR China; ^4^ Department of Pathology, Nanfang Hospital, Southern Medical University, Guangzhou, Guangdong, PR China; ^5^ Department of Emergency, Nanfang Hospital, Southern Medical University, Guangzhou, Guangdong, PR China

**Keywords:** miR-27a, PPARγ, TGF-β/Smad3, renal tubulointerstitial fibrosis, diabetic nephropathy

## Abstract

MicroRNA-27a (miR-27a) upregulation has been identified in diabetes, but the pathogenesis of miR-27a in renal tubulointerstitial fibrosis (TIF) in diabetic nephropathy (DN) has not been elucidated. Herein, we found that high glucose stimulated miR-27a expression, which directly inhibited PPARγ and promoted fibrosis in NRK-52E cells. The functional relevance of miR-27a-dependent PPARγ decrease was proven by inhibition or overexpression of miR-27a both *in vitro* and in streptozotocin-induced diabetic rats. MiR-27a, via repression of PPARγ, activates the TGF-β/Smad3 signaling and contributes to the expressional changes of connective tissue growth factor (CTGF), Fibronectin and Collagen I, key mediators of fibrosis. Furthermore, we provide evidences that plasma miR-27a upregulation contributed to unfavorable renal function and increased TIF in renal tissues of diabetic rats and DN patients. Notably, miR-27a exhibited clinical and biological relevance as it was linked to elevated serum creatinine, proteinuria, urinary N-acetyl-β-D-glucosaminidase (NAG), and reduced estimated glomerular filtration rate (eGFR). Thus, we propose a novel role of the miR-27a-PPARγ axis in fostering the progression toward more deteriorated renal TIF in DN. Monitoring plasma miR-27a level and its association with PPARγ can be used to reflect the severity of renal TIF. Targeting miR-27a could be evaluated as a potential therapeutic approach for DN.

## INTRODUCTION

Diabetes has become a major public issue worldwide. As the leading cause of end-stage renal disease (ESRD) [1], diabetic nephropathy (DN) leads to chronic renal failure and affects approximately 15–25% of type 1 diabetic patients and 30–40% of type 2 diabetic patients [2], even though numerous interventions, such as tight glycemic control, ideal control of blood pressure and blood lipid, and the rennin-angiotensin system inhibition, are extensively used. Why does the prevalence of diabetic nephropathy still remain high and why do many patients on rennin-angiotensin system inhibitors still progress to ESRD?

Fibrosis is the final common pathway of ESRD caused by chronic kidney diseases for various reasons [3]. Without doubt, how to delay the progression of fibrosis has already become hot topic for today's research. A series of studies have demonstrated that glomerular injury mainly contributes to the progression of DN. However, the pathogenic role of tubulointerstitial fibrosis (TIF) in relation to declined renal function in DN warrants exploration. In diabetes, tubules are vulnerable and injurious to various stimuli, like hypoxia, inflammation, high glucose microenvironment and immune factors [4, 5]. With time, TIF ensues, characterized by activation and proliferation of interstitial fibroblasts and connective tissue growth factors (CTGF), and by excessive synthesis and accumulation of extracellular matrix (ECM) components, including fibronectin and collagen. The mechanism is presently not clear and may be related to multiple factors such as immune, inflammation and epithelial cell transdifferentiation [6–9]. Therefore, elucidating the pathogenic mechanisms of TIF in DN is of great significance in improving the disease outcome.

Regarded as single-stranded and highly conserved small non-coding RNAs, microRNAs (miRNAs) are involved in numerous biologic processes like growth and cell proliferation, differentiation and apoptosis, by recognizing complementary sequences in the 3′-untranslated region (3′-UTR) of target mRNAs without affecting its stability [10]. MiRNA only accounts for 1–3% of human genome, but it is involved in regulating 1/3 gene expression [11] by negative regulation at the post transcriptional level. MiRNA is widely and specifically distributed in tissues and organs and has been gradually regarded as an important and early biomarker in a variety of chronic kidney diseases (CKD) [12–18]. The miR-27 family, including miR-27a and miR-27b, has emerged as a new key regulator in the physiological processes of atherosclerosis [19] and cardiovascular disease [20]. Emerging data have shown that miR-27 plays important roles in lipid metabolism, inflammation, angiogenesis, adipogenesis, oxidative stress, renin-angiotensin system, insulin resistance and type 2 diabetes [21]. MiR-27a has also been identified to represses ATP-Binding Cassette Sub-Family A Member 1 (ABCA1) function in prostate cancer [22] and in Huh-7.5 cells [23]. Dysregulation of miR-27a has been reported in different diseases such as metabolic syndrome [24], diabetes [25, 26], obesity [27], and non-alcoholic fatty liver disease [28]. Interestingly, an increase in the levels of circulating miR-27a in patients with type 1 and type 2 diabetes has been recently reported [24, 26].

Peroxisome proliferator-activated receptors (PPARs) family, consisting of PPARα, PPARβ/δ and PPARγ, plays a critical role in the regulation of multiple gene expressions. Different subtypes distribute in different tissues and possess specific regulatory function. Located at 3p25, human PPARγ widely distributes in the heart, kidney, spleen and intestine. As a member of the nuclear hormone receptor superfamily of ligand-activated transcription factors, PPARγ has been characterized to regulate adipogenesis and glucose metabolism [29]. PPARγ agonist rosiglitazone has proven effective in the treatment of diabetes and vascular diseases [30–34]. Importantly, PPARγ has been described as a negative regulator of macrophage function by suppressing the production of inflammatory cytokines [35, 36], metalloproteinases and nitric oxide [37, 38]. More recently, it has been reported that PPARγ activators inhibit transforming growth factor-β1 (TGF-β1)-induced myofibroblast transdifferentiation [39]. In diseased tissues, PPARγ expression has been shown to relate inversely with that of TGF-β1. Thus, it appears that the balance between TGF-β1 and PPARγ may determine, among other factors, whether fibrogenesis predominates after tissue injury [40]. As a pleomorphic growth factor with profibrotic properties, TGF-β1 has been implicated in many forms of natural and experimental tissue fibrosis. The profibrotic effects of TGF-β1 are mostly mediated by intracellular signals triggered by the transcription factor Smad3. TGF-β1/Smad3 signaling stimulates the expression of CTGF and epithelial-mesenchymal transition (EMT), events considered key to the development of fibrosis [41]. In fibroblasts, TGF-β1/Smad3 signaling stimulates their transdifferentiation into myofibroblasts and their expression of matrix genes like fibronectin and collagens. Considering its significance in the development of fibrosis, research directed at investigating the factors that control TGF-β1/Smad3 signaling has been intensified. Preliminary studies have suggested that PPARγ inhibits the effects of TGF-β1 via repression of Smad3. Others have demonstrated protection against TGF-β1-induced myofibroblast transdifferentiation in cells treated with PPARγ activators [42].

In terms of the relationship between miR-27a and PPARγ in diabetes, we design the present study to delve into the role of miR-27a in the progression of diabetic DN. We also explored whether the miR-27a/PPARγ signaling promotes renal TIF in DN and the underlying mechanisms.

## RESULTS

### High glucose promotes miR-27a and fibrosis via repression of PPARγ *in vitro*

**Figure 1:**
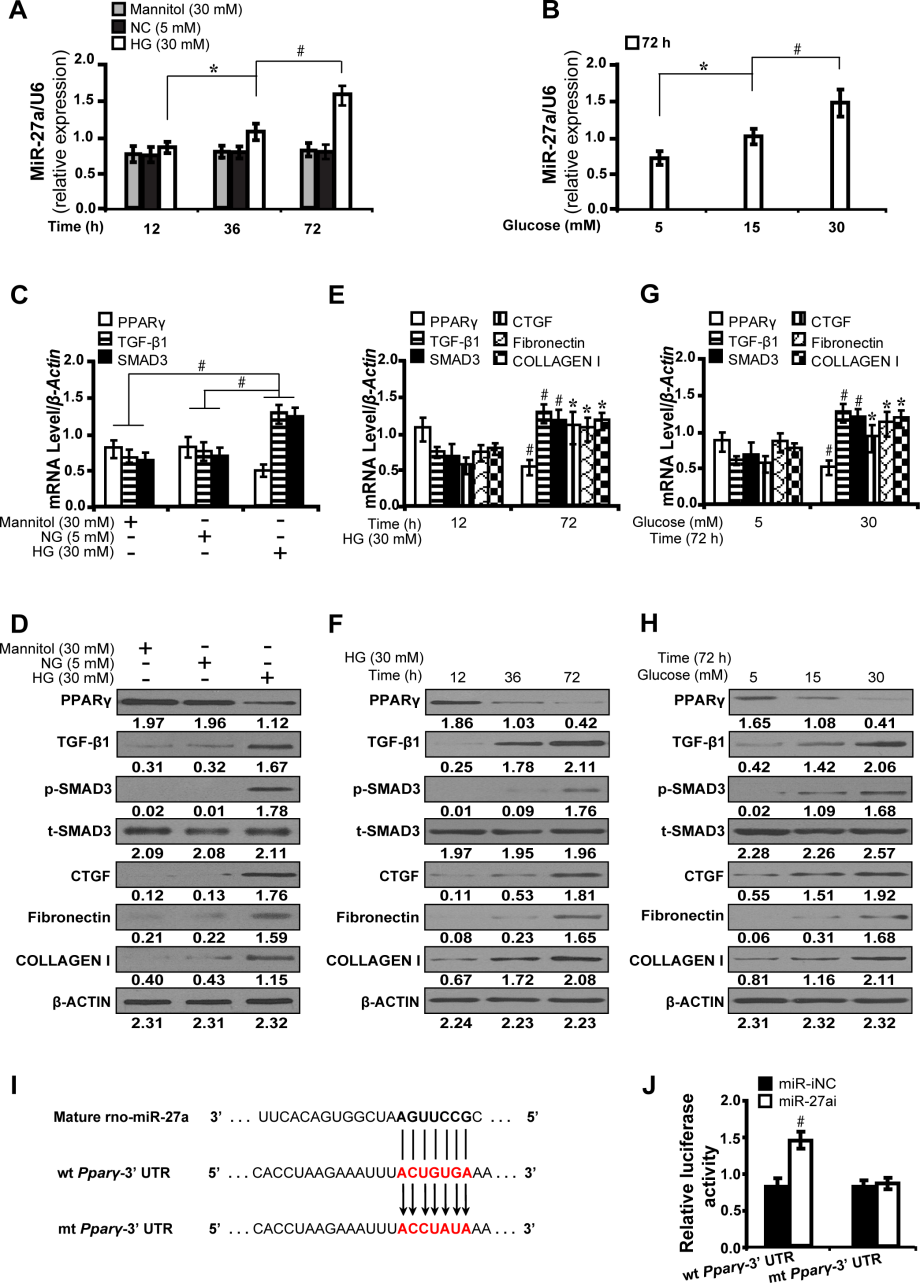
High glucose promotes miR-27a and directly modulates PPARγ-induced fibrosis *in vitro* (**A**) High glucose (30 mM) stimulated miR-27a expression in a time-dependent manner compared between groups. (**B**) Increased miR-27a level in a dose-dependent manner at 72 h. (**C**, **D**) Expression levels of *PPARγ*, TGF-β1, Smad3, CTGF, Fibronectin, and Collagen I compared between groups. (**E**, **F**) High glucose decreased the level of PPARγ but increased TGF-β1, Smad3, CTGF, Fibronectin, and Collagen I in a time and (**G** and **H**) dose dependent manner. (**I**) MiR-27a and its putative binding sequence in the 3′-UTR of PPARγ. The mutant *PPARγ* binding site was generated in the complementary site for the seed region of miR-27a. (**J**) MiR-27a inhibitor led to a noticeable increase in the luciferase activity of wt 3′-UTR of *PPARγ*. Results are presented as mean ± SD of three independent experiments. *P < 0.05; ^#^*P* < 0.001. NG, normal glucose; HG, high glucose; p-SMAD3, phospho-SMAD3; t-SMAD3, total-SMAD3; miR-iNC: miRNA inhibitor negative control; miR-27ai: miR-27a inhibitor; wt: wild type; mt: mutant type. (*n* = 6).

To investigate the effect of high glucose on the time course expression of miR-27a and downstream gene expressions, NRK-52E cells were cultured in different concentrations of glucose (5, 15 and 30 mM) for 12, 36 and 72 hours, respectively. As detected by qRT-PCR, the expression level of miR-27a increased in a time and dose dependent manner, independent of the effect of mannitol (30 mM) (Figure 1A and 1B). Next, we examined whether the changes of miR-27a expression level modulated fibrosis-related downstream gene expressions. As shown in Figure 1C and 1D, mannitol (30 mM) had no effect on the expression of PPARγ, TGF-β1 and Smad3. Furthermore, as illustrated by qRT-PCR (Figure 1E and 1G) and Western blot analyses (Figure 1F and 1H), with the increase of miR-27a, the expression level of PPARγ decreased, with concomitant increase in TGF-β1, phospho-Smad3, CTGF, Fibronectin, and Collagen I in a time and dose dependent manner. To further elucidate whether *PPARγ* is a direct target of miR-27a, we used a dual-luciferase reporter assay to detect whether miR-27a directly interacted with the 3′-UTR of *PPARγ* mRNA. It was shown that miR-27a inhibitor led to a remarkable increase in the luciferase activity of wild-type 3′-UTR of *PPARγ* but not the mutant (Figure 1I and 1J). These results suggest that miR-27a directly suppresses *PPARγ* and induces fibrosis in high glucose cultured NRK-52E cells *in vitro*.

### MiR-27a activates PPARγ-induced fibrosis in high glucose cultured NRK-52E cells

**Figure 2:**
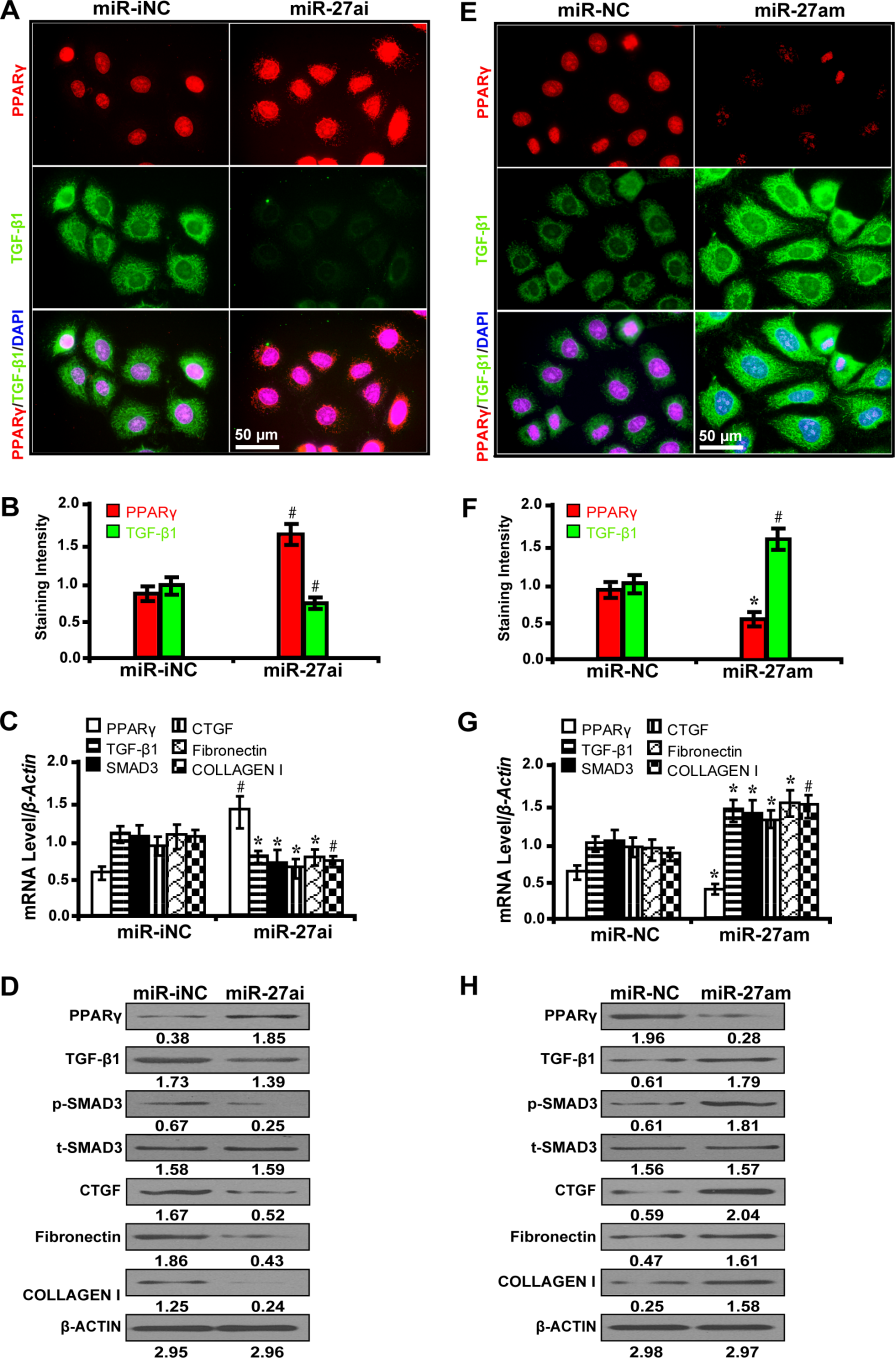
MiR-27a promotes *PPARγ*-induced fibrosis in high glucose cultured NRK-52E cells (**A**) MiR-27ai increased PPARγ expression but decreased TGF-β1 (immunofluorescence, scale bar 50 μm) and (**B**) quantification analysis. (**C**) MiR-27ai increased the expression of PPARγ but decreased TGF-β1, Smad3, CTGF, Fibronectin, and Collagen I by qRT-PCR and (**D**) Western blot analyses. (**E**) MiR-27am decreased PPARγ expression but increased TGF-β1 (immunofluorescence, scale bar 50 μm) and (**F**) quantification analysis. (**G**) MiR-27am decreased the expression of PPARγ but increased TGF-β1, Smad3, CTGF, Fibronectin, and Collagen I by qRT-PCR and (**H**) Western blot analysis. Results are presented as mean ± SD of three independent experiments. *P < 0.05; ^#^*P* < 0.001. MiR-iNC: miRNA inhibitor negative control; miR-27ai: miR-27a inhibitor; miR-NC: miRNA negative control; miR-27am: miR-27a mimic; p-SMAD3, phospho-SMAD3; t-SMAD3, total-SMAD3. (*n* = 6).

To further prove that miR-27a promotes *PPARγ*-mediate fibrosis *in vitro*, we examined the effect of miR-27a inhibitor and mimics on fibrosis-related downstream gene expressions in high glucose (30 mM) cultured NRK-52E cells. The results have shown that miR-27a inhibitor (miR-27ai) caused increased expression of PPARγ but decreased TGF-β1 as detected by immunofluorescence microscopy (Figure 2A and 2B). Accordingly, miR-27a inhibition led to increased PPARγ expression but decreased expression of TGF-β1, Smad3, CTGF, Fibronectin, and Collagen I by qRT-PCR (Figure 2C) and Western blot analyses (Figure 2D). In contrast, miR-27a enrichment with miR-27a mimics (miR-27am) had the opposite effects (Figure 2E, 2F, 2G and 2H). These data demonstrate that miR-27a, through repression of PPARγ, induces fibrosis *in vitro*.

### PPARγ alleviates TGF-β/SMAD3-induced fibrosis in high glucose cultured NRK-52E cells

**Figure 3:**
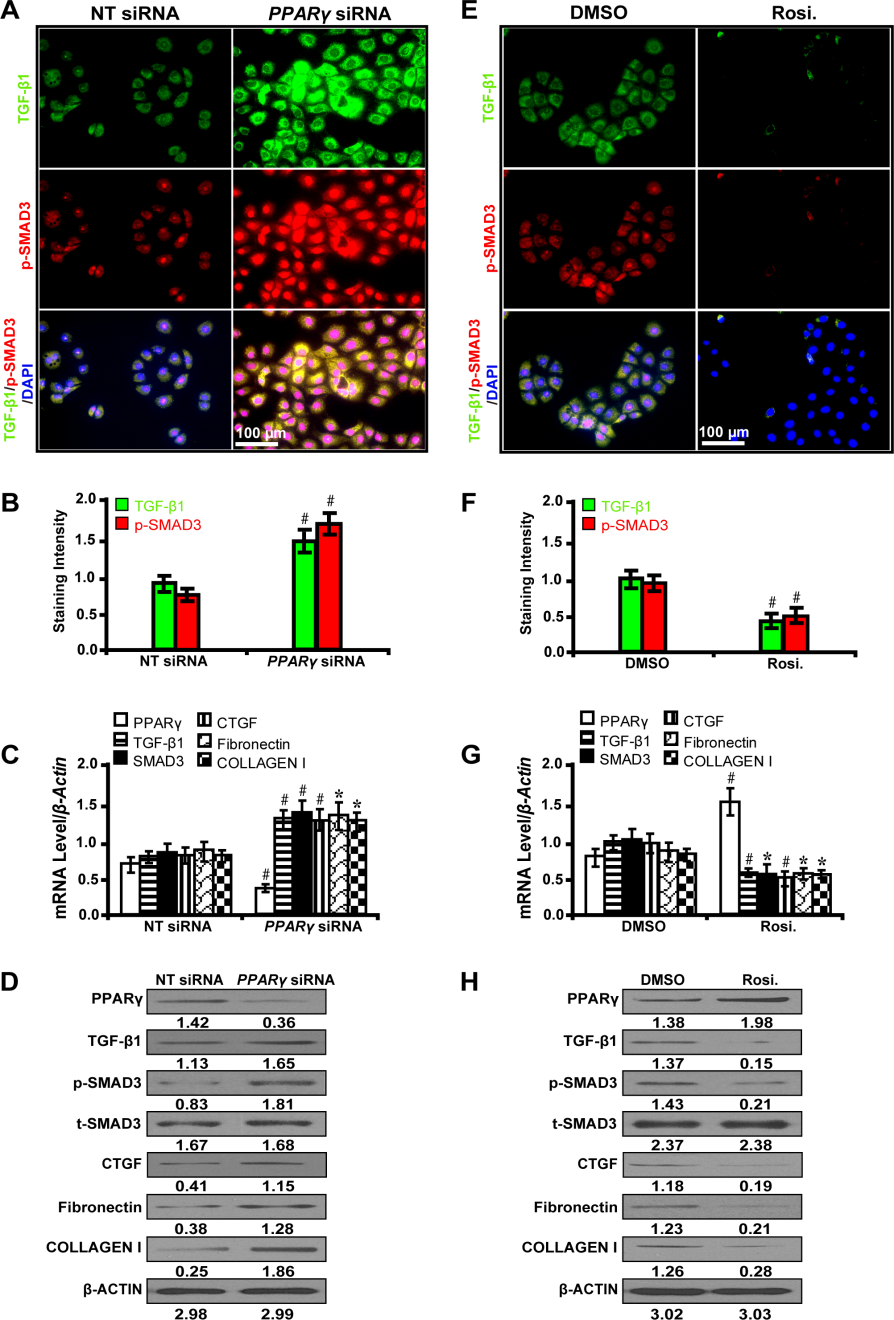
PPARγ attenuates TGF-β/SMAD3-induced fibrosis in high glucose cultured NRK-52E cells (**A**) PPARγ siRNA up-regulated the expression of TGF-β1 and p-SMAD3 (immunofluorescence, scale bar 50 μm) and (**B**) quantification analysis. (**C**) PPARγ siRNA decreased the expression of PPARγ but increased TGF-β1, Smad3, CTGF, Fibronectin, and Collagen I by qRT-PCR and (**D**) Western blot analyses. (**E**) Rosiglitazone downregulated the expression of TGF-β1 and p-SMAD3 (immunofluorescence, scale bar 100 μm) and (**F**) quantification analysis. (**G**) Rosiglitazone increased the expression of PPARγ but decreased TGF-β1, Smad3, CTGF, Fibronectin, and Collagen I by qRT-PCR and (**H**) Western blot analyses. Results are presented as mean ± SD of three independent experiments. *P < 0.05; ^#^*P* < 0.001. NT: non-targeting; siRNA: small interfering RNA; Rosi.: rosiglitazone; p-SMAD3, phospho-SMAD3; t-SMAD3, total-SMAD3. (*n* = 6).

To decipher whether PPARγ mitigates fibrosis through the TGF-β pathway, we treated NRK-52E cells with PPARγ siRNA and its agonist rosiglitazone. It has been shown that PPARγ silencing with siRNA significantly upregulated the expression level of TGF-β1 and phospho-SMAD3 as detected by immunofluorescence microscopy (Figure 3A and 3B). Furthermore, PPARγ siRNA increased the expression of CTGF, Fibronectin, and Collagen I by qRT-PCR (Figure 3C) and Western blot analyses (Figure 3D). Conversely, PPARγ agonist rosiglitazone exerted the opposite effects (Figure 3E, 3F, 3G and 3H). These results indicate that PPARγ attenuates fibrosis through suppression of the TGF-β/SMAD3 signliang in high glucose cultured NRK-52E cells *in vitro*.

### Requirement of PPARγ for the miR-27a antagonism effect on downstream gene expressions *in vitro*

**Figure 4:**
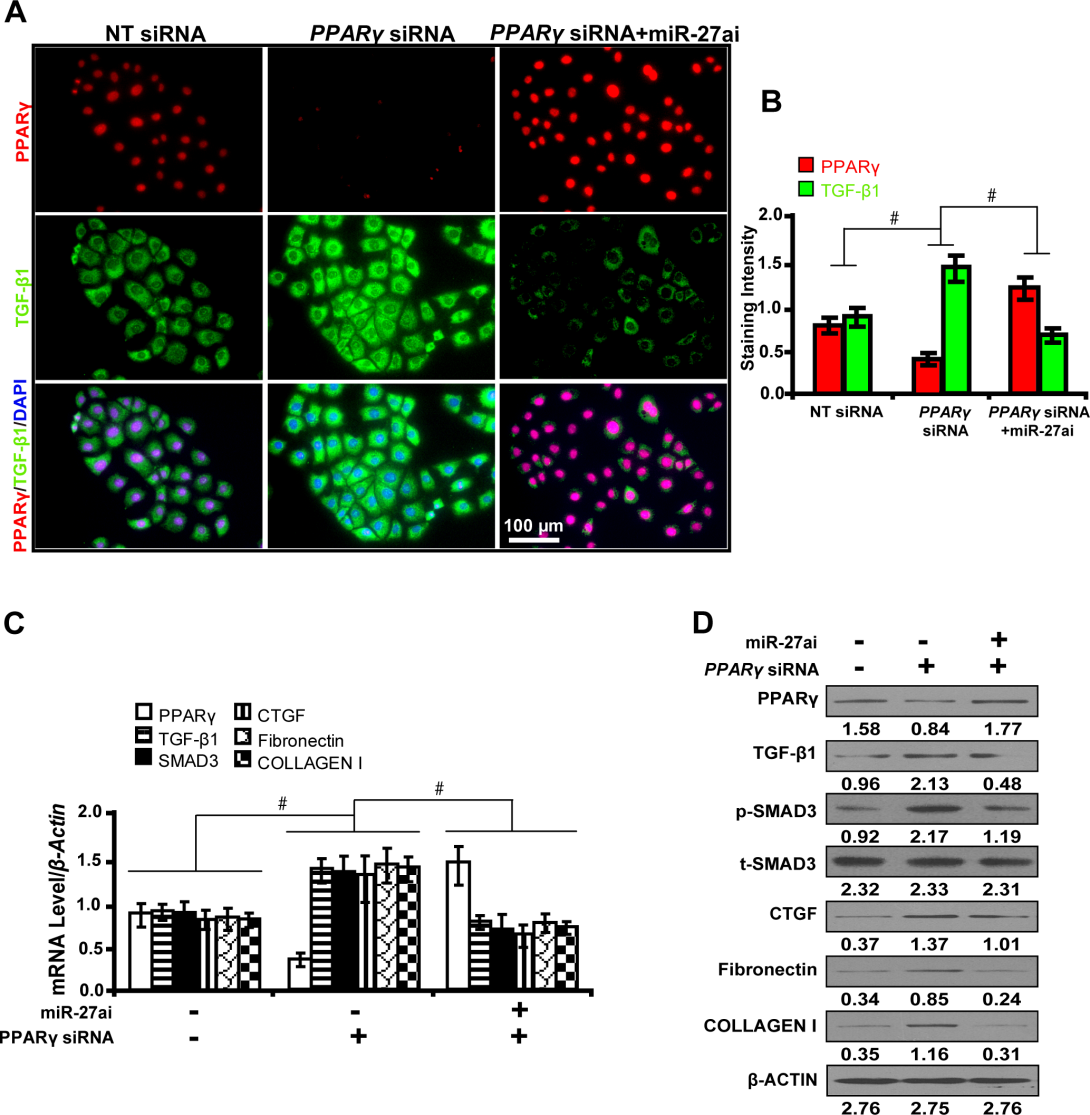
Requirement of PPARγ for the miR-27a antagonism effect on downstream gene expressions *in vitro* (**A**) MiR-27ai attenuated the silencing effect of PPARγ siRNA on TGF-β1 expression (immunofluorescence, scale bar 100 μm) and (**B**) quantification analysis. (**C**) The restoration of PPARγ decreased the expression of TGF-β1, Smad3, CTGF, Fibronectin, and Collagen I by qRT-PCR and (**D**) Western blot analyses. Results are presented as mean ± SD of three independent experiments. *P < 0.05; ^#^*P* < 0.001. NT: non-targeting; siRNA: small interfering RNA; miR-27ai: miR-27a inhibitor; p-SMAD3, phospho-SMAD3; t-SMAD3, total-SMAD3. (*n* = 6).

In order to explore whether the effect of miR-27a on downstream gene expressions depended on *PPARγ*, cells were first treated with *PPARγ* siRNA and then with miR-27a inhibitor. As shown by immunofluorescence microscopy (Figure 4A) and quantification of the staining intensity (Figure 4B), upon *PPARγ* silencing, TGF-β1 expression was significantly increased. However, when we treated *PPARγ*-silenced cells with miR-27a inhibitor, we found that the expression of PPARγ was restored. Concurrently, the restoration of PPARγ led to decreased expression of TGF-β1, Smad3, CTGF, Fibronectin, and Collagen I, downstream genes related with fibrosis, as detected by qRT-PCR (Figure 4C) and Western blot analyses (Figure 4D). These results suggest that *PPARγ*, as a direct target of miR-27a, decreases fibrosis related gene expressions *in vitro*.

### MiR-27a depletion upregulates PPARγ and inhibits fibrosis *in vivo*

To investigate the biological function of miR-27a *in vivo*, we administered miR-27a inhibitor to diabetic rats. We found that inhibition of miR-27a significantly decreased the level of serum creatinine (Scr), serum blood urea nitrogen (BUN), urinary N-acetyl-β-D-glucosaminidase (NAG), urine albumin excretion rate (UAER), urine albumin to creatinine ratio (UACR) and elevated creatinine clearance rate (Ccr) (Table 1). The mRNA level of miR-27a increased in the plasma (Figure 5A) and kidney tissues (Figure 5B) of diabetic rats but was decreased with miR-27a inhibition. Furthermore, inhibiting miR-27a elevated the expression level of PPARγ but downregulated TGF-β1, Smad3, CTGF, Fibronectin, and Collagen I expression by qRT-PCR (Figure 5C), Western blot (Figure 5D) and immunohistochemistry analyses (Figure 5E). Correspondingly, TIF was improved in diabetic rats treated with miR-27a inhibitor as detected by Masson's trichrome stain and the quantification analysis (Figure 5F).

**Table 1:** Biological parameters for diabetic rats treated with miR-27a inhibitor at week 12

Variables	DM_miR-iNC (*n* = 7)	DM_miR-27ai (*n* = 7)
Scr (umol/L)	114.37 ± 7.56	67.25 ± 2.28*
Serum BUN (mmol/L)	15.63 ± 4.24	11.05 ± 1.52*
Blood glucose (mmol/L)	26.31 ± 1.36	25.70 ± 2.15
Urinary NAG (U/L)	35.37 ± 5.02	16. 38 ± 3.23^#^
UAER (ug/min)	1.45 ± 0.21	0.67 ± 0.06^#^
UACR (ug/mmol)	27.32 ± 2.32	15.35 ± 1.16^#^
Ccr (mL·min^−1^·Kg^−1^)	3.48 ± 0.45	7.37 ± 0.82^#^

DM_miR-iNC, diabetic rats treated with miRNA inhibitor negative control; DM_miR-27ai, diabetic rats treated with miR-27a inhibitor; Scr, serum creatinine; Serum BUN, serum blood urea nitrogen; Urinary NAG, urinary N-acetyl-β-D-glucosaminidase; UAER, urine albumin excretion rate; UACR, urine albumin to creatinine ratio; Ccr, creatinine clearance rate;

* *P* < 0.01;

# *P* < 0.001.

**Figure 5:**
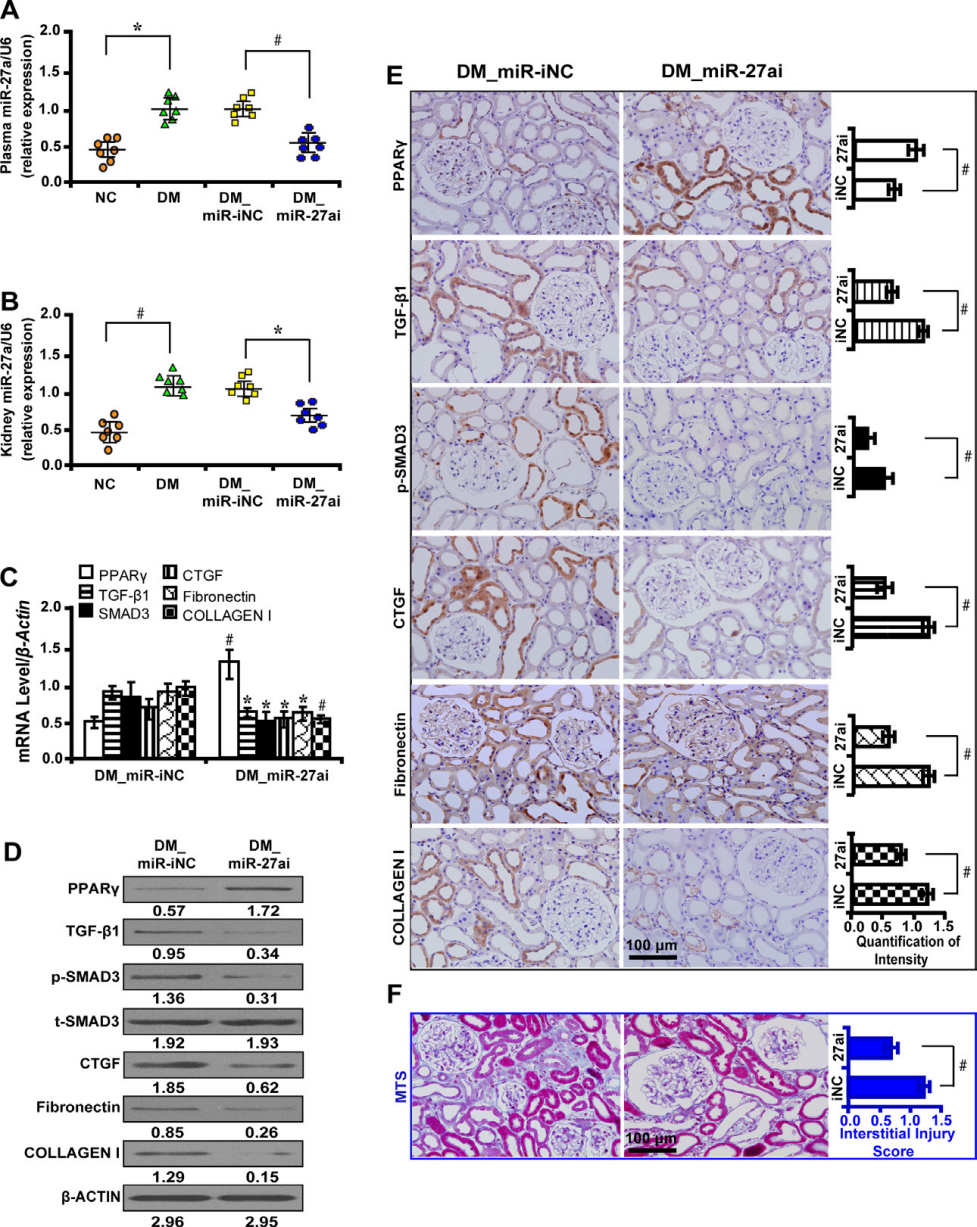
MiR-27a inhibitor improves fibrosis *in vivo* (**A**) The miR-27a level was increased in the plasma and (**B**) kidney tissues of diabetic rats and decreased with miR-27a inhibition treatment. (**C**) MiR-27a inhibitor increased PPARγ expression but decreased TGF-β1, Smad3, CTGF, Fibronectin, and Collagen I by qRT-PCR, (**D**) Western blot and (**E**) immunohistochemistry analyses (scale bar 100 μm). (**F**) Improved tubulointerstitial fibrosis in diabetic rats treated with miR-27a inhibitor by Masson's trichrome stain (scale bar 100 μm) and the quantification analysis. Results are presented as mean ± SD of three independent experiments. *P < 0.05; ^#^*P* < 0.001. NC, normal control; DM, diabetes mellitus; DM_miR-iNC, diabetic rats treated with miRNA inhibitor negative control; DM_miR-27ai, diabetic rats treated with miR-27a inhibitor; p-SMAD3, phospho-SMAD3; t-SMAD3, total-SMAD3; MTS, Masson' s trichrome stain. (*n* = 7).

### MiR-27a mimics promote fibrosis via PPARγ pathway *in vivo*

We treated diabetic rats with miR-27a mimics and found that the level of Scr, serum BUN, urinary NAG, UAER, UACR were significantly increased and Ccr was decreased (Table 2). The mRNA level of miR-27a increased in the plasma (Figure 6A) and kidney tissues (Figure 6B) of diabetic rats and further increased with miR-27a mimics treatment. Moreover, miR-27a mimics downregulated the expression of PPARγ but increased TGF-β1, Smad3, CTGF, Fibronectin, and Collagen I expression by qRT-PCR (Figure 6C), Western blot (Figure 6D) and immunohistochemistry analyses (Figure 6E). TIF was worsened in diabetic rats treated with miR-27a mimics as detected by Masson's trichrome stain and the quantification analysis (Figure 6F).

**Table 2:** Biological parameters for diabetic rats treated with miR-27a mimics at week 12

Variables	DM_miR-NC (*n* = 7)	DM_miR-27am (*n* = 7)
Scr (umol/L)	112.24 ± 6.38	122.30 ± 7.26*
Serum BUN (mmol/L)	16.12 ± 4.06	19.27 ± 2.62*
Blood glucose (mmol/L)	27.52 ± 0.76	25.54 ± 1.85
Urinary NAG (U/L)	37.26 ± 6.58	58. 36 ± 7.35^#^
UAER (ug/min)	1.48 ± 0.25	2.85 ± 0.92^#^
UACR (ug/mmol)	25.65 ± 2.18	58.67 ± 3.34^#^
Ccr (mL·min^−1^·Kg^−1^)	3.12 ± 0.34	0.68 ± 0.05^#^

DM_miR-NC, diabetic rats treated with miRNA mimics negative control; DM_miR-27am, diabetic rats treated with miR-27a mimics; Scr, serum creatinine; Serum BUN, serum blood urea nitrogen; Urinary NAG, urinary N-acetyl-β-D-glucosaminidase; UAER, urine albumin excretion rate; UACR, urine albumin to creatinine ratio; Ccr, creatinine clearance rate;

* *P* < 0.01;

# *P* < 0.001.

**Figure 6:**
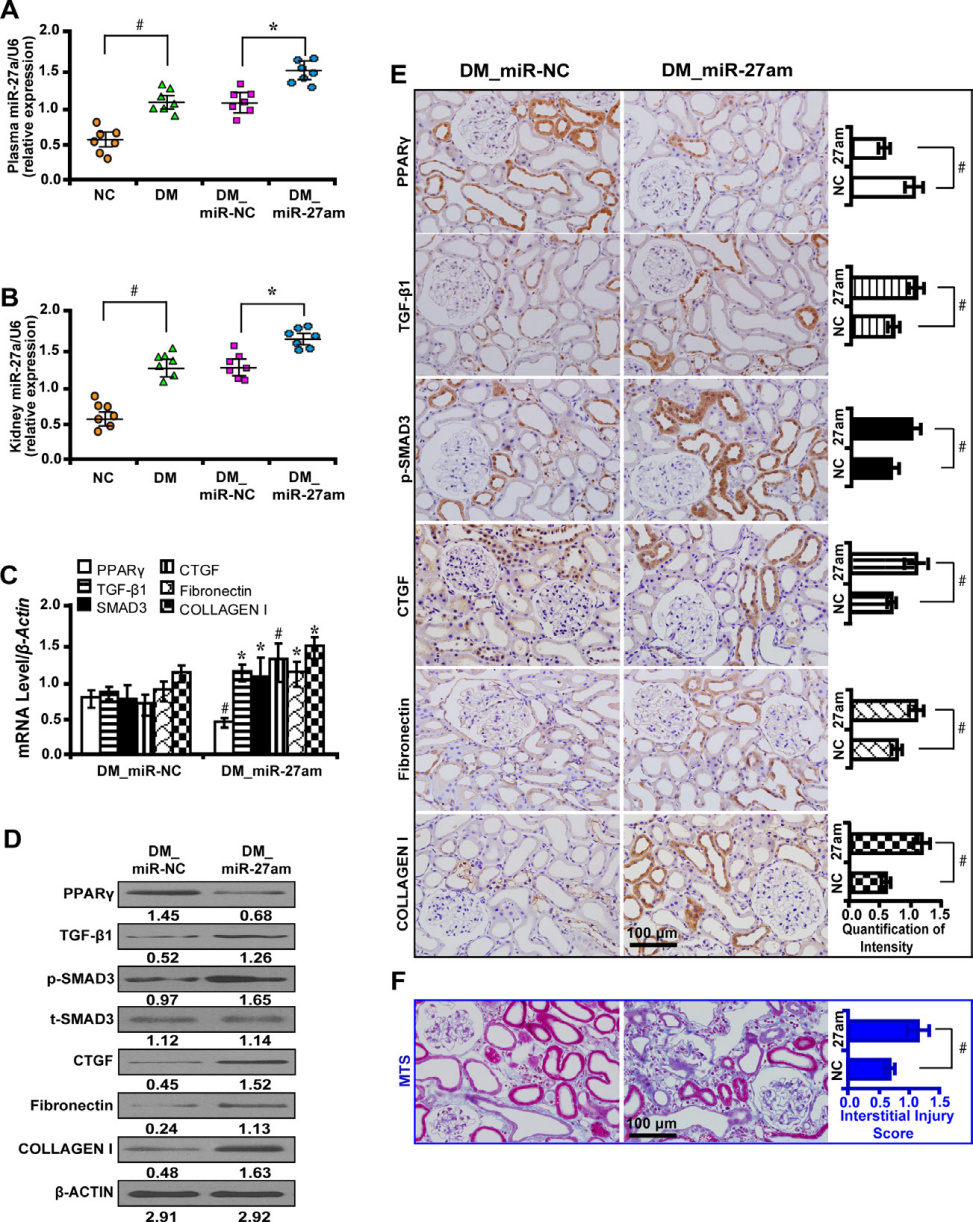
MiR-27a mimics aggravate fibrosis *in vivo* (**A**) The miR-27a level was increased in the plasma and (**B**) kidney tissues of diabetic rats and further increased treated with miR-27a mimics. (**C**) MiR-27a mimics decreased PPARγ expression but increased TGF-β1, Smad3, CTGF, Fibronectin, and Collagen I by qRT-PCR, (**D**) Western blot and (**E**) immunohistochemistry analyses (scale bar 100 μm). (**F**) Worsened tubulointerstitial fibrosis in diabetic rats treated with miR-27a mimics by Masson's trichrome stain (scale bar 100 μm) and the quantification analysis. Results are presented as mean ± SD of three independent experiments. *P < 0.05; ^#^*P* < 0.001. NC, normal control; DM, diabete mellitus; DM_miR-NC, diabetic rats treated with miRNA negative control; DM_miR-27am, diabetic rats treated with miR-27a mimics; p-SMAD3, phospho-SMAD3; t-SMAD3, total-SMAD3; MTS, Masson' s trichrome stain. (*n* = 7).

### Elevated plasma miR-27a reflects unfavorable renal function and increased tubulointerstitial fibrosis in patients with diabetic nephropathy

**Figure 7:**
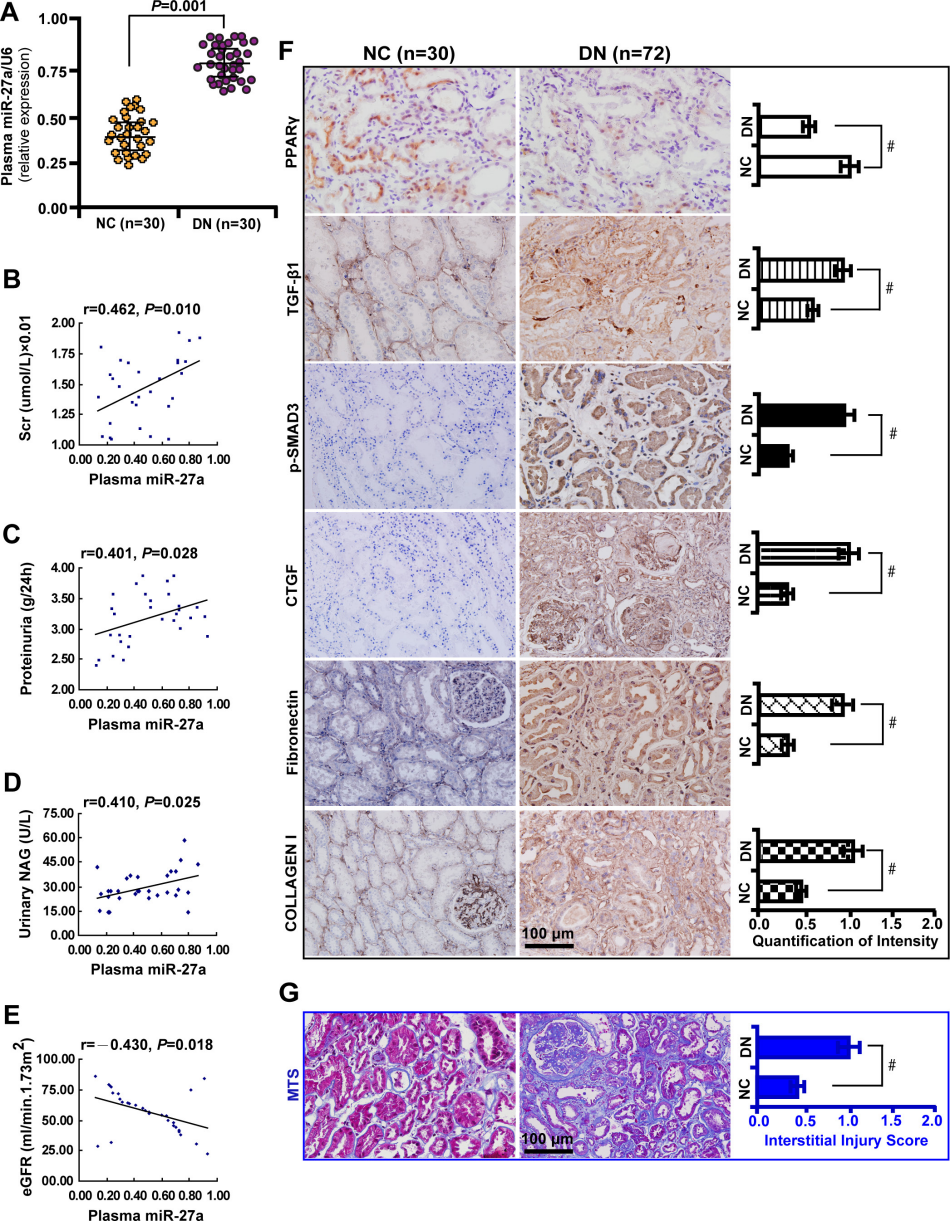
Elevated plasma miR-27a reflects unfavorable renal function and increased tubulointerstitial fibrosis in DN patients (**A**) Increased plasma miR-27a level in DN patients (*n* = 30) compared with healthy normal controls (*n* = 30). (**B**) Positive correlation between plasma miR-27a and serum creatinine, (**C**) proteinuria, (**D**) urinary NAG. (**E**) Negative correlation between plasma miR-27a and eGFR. (**F**) Decreased PPARγ expression and increased TGF-β1, p-Smad3, CTGF, Fibronectin, and Collagen I in renal biopsies of DN patients (*n* = 72) by immunohistochemistry (scale bar 100 μm) and quantification of the staining intensity. (**G**) Worsened tubulointerstitial fibrosis in DN compared with normal controls by Masson' s trichrome stain (scale bar 100 μm) and the quantification analysis. Results are presented as mean ± SD of three independent experiments. *P < 0.05; ^#^*P* < 0.001. NC, normal control; DN, diabetic nephropathy; Scr, serum creatinine; NAG, N-acetyl-β-D-glucosaminidase (NAG); eGFR, estimated glomerular filtration rate; p-SMAD3, phospho-SMAD3; MTS, Masson' s trichrome stain.

**Figure 8:**
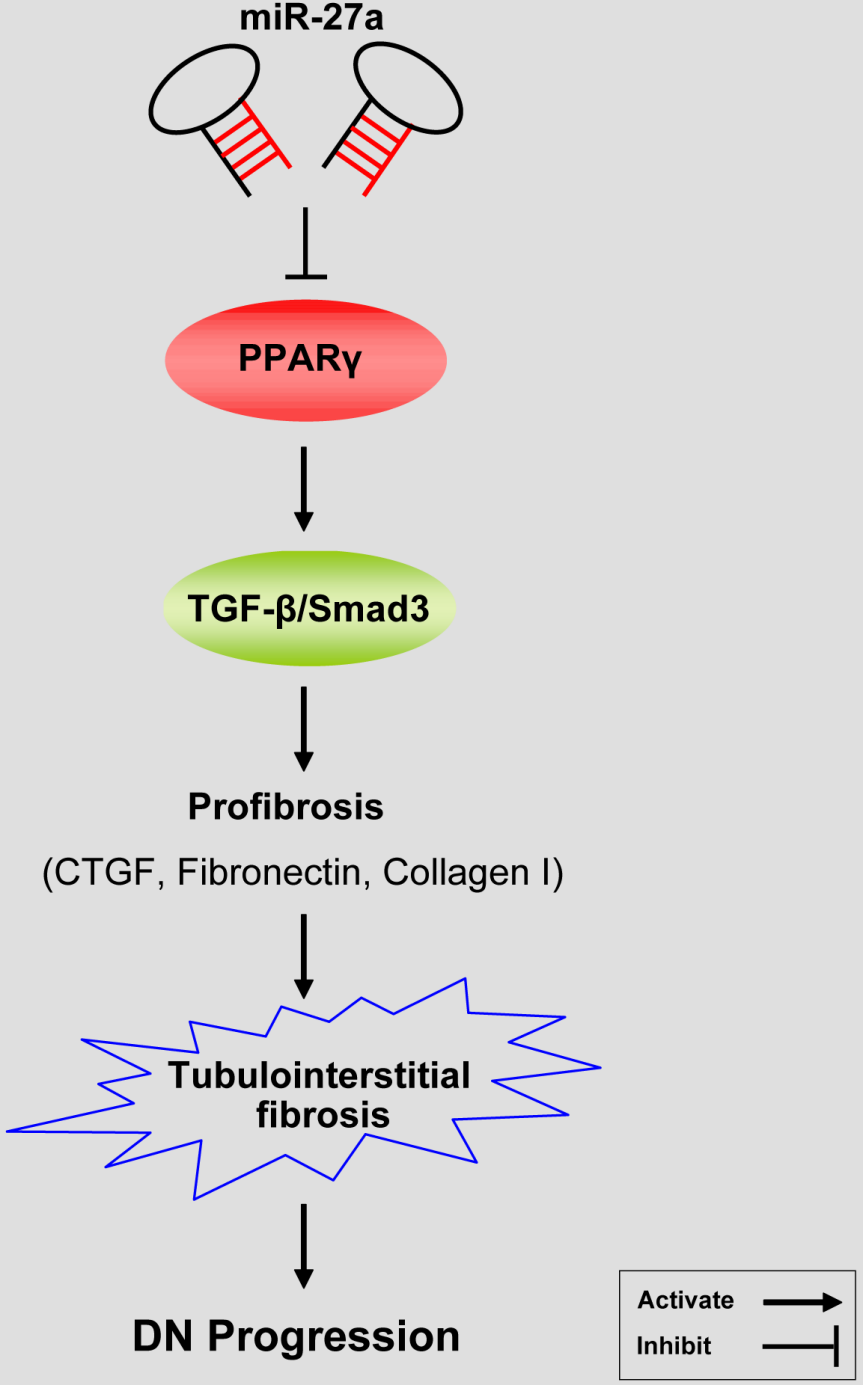
A hypothetical model illustrating that miR-27a/PPARγ signaling regulated renal tubulointerstitial fibrosis through the TGF-β1/Smad3 mediated fibrosis in diabetic nephropathy MiR-27a inhibits PPARγ and aggravates tubulointerstitial fibrosis and progression of DN through activating TGF-β1/Smad3 signaling pathway and promoting the expression of CTGF, Fibronectin, and Collagen I.

To explore the clinical significance of miR-27a in DN patients, we analyzed the correlation between serum miR-27a level with biological parameters of DN patients. We found that the level of serum miR-27a of DN patients was increased compared with healthy normal controls (Figure 7A). In DN patients, the level of serum miR-27a was positively correlated with serum creatinine (Figure 7B), proteinuria (Figure 7C), urinary NAG (Figure 7D) and negatively with eGFR (Figure 7E). It was shown by immunohistochemistry (Figure 7F) and quantification of the staining intensity (Figure 7G) that the protein level of PPARγ was decreased with concomitant increase in the level of TGF-β1, phospho-Smad3, CTGF, Fibronectin, and Collagen I in renal biopsies of DN patients. Moreover, TIF was exacerbated in DN compared with normal controls as detected by Masson' s trichrome stain and the quantification analysis (Figure 7G). These data further validate the *in vitro* and *in vivo* results that miR-27a confers unfavorable renal function and TIF through PPARγ-induced activation of the TGF-β1/Smad3 pathway. A hypothetical model illustrated that miR-27a/PPARγ signaling promoted renal TIF through the TGF-β1/Smad3-induced fibrosis in DN (Figure 8).

## DISCUSSION

The present study demonstrates the significant role of miR-27a/PPARγ pathway in renal TIF in DN. We found that miR-27a antagonized the expression of PPARγ and promoted TIF through the TGF-β1/Smad3 signaling. Moreover, we provide evidences that PPARγ, as a direct target of miR-27a, suppresses TGF-β1-induced fibrosis through inactivating fibrosis-related gene expressions. Collectively, the current results implicate miR-27a/PPARγ as an initiating mechanism triggering the process of renal TIF in DN by activating the TGF-β1/Smad3 signaling [43].

Our data also revealed that in renal biopsy tissues of DN, tubular expression of PPARγ were decreased but TGF-β1, Smad3, CTGF, Fibronectin and Collagen I increased with concurrent deterioration of fibrosis. Similar results were found in kidney tissues of diabetic rats. Furthermore, the mRNA level of miR-27a increased in the serum of diabetic patients, accompanied by increased Scr, proteinuria, urinary NAG and decreased eGFR. These results suggest that in DN, miR-27a confers an unfavorable renal function and serum miR-27a might serve as an alternative approach to reflect the severity of renal tubulointerstitial fibrosis.

We found that miR-27a directly targets *PPARγ* and promotes TGF-β1-induced expression of profibrotic genes in DN. Previous studies have reported that miR-27a targets *PPARγ* in mediating inflammatory processes [21]. MiR-27a has also been shown to repress the activity of *PPARγ* in the pulmonary vasculature [44] and adipocyte differentiation [45]. MiR-27a and miR-27b are negative regulatory factors of adipocytes and have been shown to directly target PPARγ [46, 47]. These findings provide evidences that PPARγ is a critical intermediate modulator downstream of miR-27a. Our current study demonstrates that miR-27a promotes renal TIF in DN through repression of PPARγ. We also found that PPARγ inhibits TGF-β1/Smad3 signaling in both high glucose cultured tubular epithelial cells and diabetic rats. The effectiveness of PPARγ agonist rosiglitazone in the treatment of DN has been acknowledged [48], however, the mechanisms of which needs further exploration. The present result adds further evidences that PPARγ improves DN by relieving TIF. By targeting PPARγ, miR-27a serves as an initiator in triggering a cascade of several pro-fibrotic mediators relevant in the development of fibrosis in DN.

The miRNAs exert actions in different tissues, while circulating miRNAs hold much promise as biomarkers of type 2 diabetes. The present finding indicates that circulating miRNAs hold much potential as biomarkers for the progression, grading and prognosis of type 2 diabetes [49]. The miRNAs that circulate in the blood are in a stable form and remain stable even after multiple freeze-thaw cycles. They can be detected by minimally invasive techniques and are specific to tissue and disease states. If these miRNAs in circulating blood can serve as biomarkers, they would provide a minimally invasive biomarker approach that would be extremely useful in diagnosing and monitoring type 2 diabetes. The present study has demonstrated that plasma miR-27a is increased in DN and confers declined renal function. However, more studies are needed to verify the value of circulating miRNAs in DN.

TIF is characterized by increased deposition of ECM, represented with increased expression of laminin, type I and IV collagen, CTGF and fibronectin [50]. TIF not only accounts for declined renal function, but contributes to the progression of CKD [51, 52]. ECM is mainly secreted and synthesized by fibroblasts/myofibroblasts in glomeruli and renal tubular interstitium. A recent study has demonstrated that bone marrow-derived macrophage-myofibroblast transition (MMT) contributes to the production of ECM [8]. However, the origin of fibroblasts/myofibroblasts in DN remains unclear and needs further elucidation.

Multiple studies have shown that PPARγ is a negative regulator in the fibrosis pathway and inhibits the deposition of ECM in DN [53, 54]. In order to further decipher the protective effect of PPARγ on TIF, we silenced PPARγ gene and observed its downstream gene expressions. Our results have shown that silencing PPARγ significantly increased the expression level of CTGF, Fibronectin and collagen I through upregulating TGF-β1/Smad3 signaling. Similar results were obtained when PPARγ agonist was used. These results demonstrate that PPARγ is a critical factor in inhibiting TIF, in agreement with previous studies showing the critical role of PPARγ in fibrosis [55, 56]. However, further studies are still needed to investigate whether TGF-β1-induced myofibroblast formation/transdifferentiation contributes to renal TIF in DN.

We did not analyze the level of miR-27a in different stages of DN, which limited its clinical value in predicting the progression of DN. Future studies are directed to explore the differential changes of miR-27a in DN patients with only large vascular complications versus patients without target organ damage. In doing so, we could be able to find out the correlation between the level of miR-27a and the severity of DN. Meanwhile, further studies are warranted to validate the significance of miR-27a in expanded samples of DN and also in other non-renal diseases. The pathophysiological mechanism of TIF is very complex and it is also possible that other fibrogenic pathways may also contribute in part to TIF in DN progression, which awaits further in-depth study.

In conclusion, our data demonstrate a mechanism that the miR-27a/PPARγ pathway promotes TIF in DN through the TGF-β1/Smad3 pathway. Serum miR-27a is a useful biomarker in evaluating the severity of renal TIF in DN. Targeting miR-27a might be an alternative to suppress renal TIF in DN and opens new avenues into novel therapeutic strategies.

## MATERIALS AND METHODS

### Cell culture studies

Rat kidney tubular epithelial cells (NRK52E; American Type Culture Collection, Rockville, MD) were cultured in high glucose (30 mM) for one week, supplemented with Dulbecco's Modified Eagle's medium (DMEM)/F-12 containing 10% fetal bovine serum (FBS), penicillin (200 U/ml), and streptomycin (200 μg/ml) (Gibco BRL, Grand Island, NY). NRK52E cells were grown to 80%–90% confluence and made quiescent by incubation overnight in a serum-free medium before experimentation.

### Luciferase reporter assay

The predicted 3′-untranslated regions (UTR) sequence of PPARγ interacting with miR-27a and mutated sequences within the predicted target sites were synthesized and inserted into the pRL-TK control vector (Promega, Madison, WI, USA). NRK52E cells transfected with 120 ng miR-27a inhibitor or negative controls, followed by co-transfection with 30 ng of the wild-type or mutant 3′-UTR of PPARγ using 0.45 μL of Fugene (Promega, Madison, WI, USA). Luciferase assay was carried out on extracts from the cells 48 hours post transfection and measured using Dual-Luciferase Assay System (Promega, Madison, WI, USA). pRL-TK expressing Renilla luciferase was co-transfected as an internal control. Data were normalized by the ratio of Firefly and Renilla luciferase activities.

### Transfection of miRNA mimics, inhibitors and small interfering RNA

MiR-27a inhibitor (miR-27ai), miR-27a mimics (miR-27am), or the appropriate negative controls (NC) of miRNA inhibitor (miR-iNC) and miRNA mimics (miR-NC), respectively, were purchased from GenePharma (Shanghai, China) and transfected at a final concentration of 50–100 nM in the cells using HiPerFect Transfection Reagent (Qiagen, Hilden, Germany) according to the manufacturer's recommendations. Expression of murine PPARγ was knocked down with small interfering RNA (siRNA) duplexes using Oligofectamine (Invitrogen, Carlsbad, CA). The target sequence for *PPARγ* mRNA was: 5′-AAUAUGACCUGAAGCUCCAAGAAUAAG-3′. Non-targeting siRNA pool (D-001206–13–05; Dharmacon, Fisher Scientific, Pittsburgh, PA) was used as a negative control. Cells were transfected with 1 μg of siRNA in reduced serum medium (OPTI-MEM-I; Invitrogen, Carlsbad, CA) according to the manufacturer's protocol and harvested 72 hours post transfection. The RNA and protein were extracted and analyzed, respectively.

### Drug treatment

PPARγ agonist rosiglitazone (Rosi.; 50 nM; R2408; Sigma-Aldrich, St. Louis., MO) was used to treat NRK52E cells. Cells in each group were treated for 72 hours and then harvested for further analyses.

### Animal studies

The study protocols conform to the Guide for the Care and Use of Laboratory Animals published by the US National Institutes of Health (NIH Publication No. 85–23, revised 1996) and was approved by the Animal Ethics Committee at Nanfang Hospital, Southern Medical University, Guangzhou, China. Male Sprague Dawley rats (6–8 weeks of age) were kept in the Animal Center of Nanfang Hospital according to the policy of the Committee for Animal Usage. Diabetes was induced with streptozotocin (65mg/kg; S0130; Sigma-Aldrich, St. Louis., MO) according to the protocol described previously [57, 58]. To investigate the effect of miR-27a on renal tubulointerstitial fibrosis, miR-27a inhibitor or mimics (4 ng/mm^3^) was injected peritoneally to diabetic rats every day. The appropriate scrambled RNAs were included as controls.

Two groups with seven rats each were studied: diabetic rats treated with dimethyl sulphoxide (DMSO) and diabetic rats treated with miR-27a inhibitor or mimics. No adverse or toxic effects were observed. Blood glucose level was measured every week. The treatment continued until the rats were euthanized. Twelve weeks after the streptozotocin injection, blood was drawn from the tail vein and plasma samples were prepared for analyzing creatinine, BUN and glucose level. Rats were housed in metabolic cages and urine was collected for the determination of NAG, UAER and UACR. Blood glucose, serum BUN, serum and urine creatinine were analyzed using a Beckman Coulter AU480 Chemistry Analyzer (Beckman, USA). Urine albumin was determined with an ELISA kit specific for rat albumin (E111–125, Bethyl Laboratories, Montgomery, TX). Urinary NAG was determined by an assay kit (Jiancheng, Nanjing, China) that used an enzymatic colorimetric method read by a microplate reader (Epoch, BioTek, USA). Ccr was calculated as urinary creatinine (umol/L) × urine volume (mL/min)/serum creatinine (umol/L), and was expressed as mL·min^−1^·Kg^−1^. All experiments were repeated in triplicate.

At 12 weeks after the induction of diabetes, rats were anesthetized with pentobarbital sodium (P3761, 30 mg/kg, Sigma-Aldrich, St. Louis., MO). Left kidneys were obtained and fixed in 10% formalin in phosphate buffered saline (PBS) for 24 hours and embedded in paraffin for histological analysis. The right kidney was snap-frozen and stored at −80°C for further analysis.

### Laser capture microdissection (LCM)

Frozen kidney tissues from normal control and diabetic rats were cut at 8 μm thickness and renal tubules and interstitium were microdissected using the PALM MicroBeam LCM system (Zeiss, Germany) according to the manufacturer's instructions.

### Quantitative real-time reverse transcription-PCR analysis

Total RNA from NRK-52E cells and microdissected renal tubules were extracted using TRIzol reagent (MRC, Cincinnati, OH). First strand cDNA was synthesized using 2 μg of total RNA treated with Moloney murine leukemia virus reverse transcriptase (Promega, Madison, WI) according to the manufacturer's instructions. Quantitative real-time reverse transcription-PCR (RT-PCR) analysis was performed in triplicate with Power PCR SYBR Green Master Mix (Applied Biosystems, Carlsbad, CA) using the ABI PRISM 7500 FAST Real-TIME PCR System (Applied Biosystems, Carlsbad, CA) with results normalized to β-actin expression. The ΔΔCT method was used to calculate relative expression. Primer sequences used in RT-PCR are shown in Table 3.

**Table 3:** Primer sets used in real time RT-PCR

Genes (rat)	Forward Primer	Reverse Primer
*Pparγ*	5′-CGCAGCCTCAGCCAAGAC-3′	5′-TGGGGAGAGAGGACAGATGG-3′
*TGF-β1*	5′-GACTCTCCACCTGCAAGACC-3′	5′-GGACTGGCGAGCCTTAGTTT-3′
*Smad3*	5′-AACTGCAGTGCCGCTATCC-3′	5′-CGCCCGAACTTCGCTTTTAAC-3′
*CTGF*	5′-CACCCGGGTTACCAATGACA-3′	5′-TTCATGATCTCGCCATCGGG-3′
*Fibronectin*	5′-CCCAATTGAGTGCTTCATGCC-3′	5′-AACTCCCAGGGTGATGCTTG-3′
*Collagen I*	5′-GATGGACTCAACGGTCTCCC-3′	5′-CGGCCACCATCTTGAGACTT-3′
*β-actin*	5′-ATGATGATATCGCCGCGCTC-3′	5′-TCGATGGGGTACTTCAGGGT-3′

To assess the level of miRNA expression, total RNA extracted from the cells, human plasma samples, rat kidney tissues or plasma samples was reversely transcribed into cDNA using miRScript PCR System and then analyzed by qRT-PCR with the miScript SYBR Green PCR Kit using the specific miR-27a miScript Primer Assays (Qiagen, Hilden, Germany) according to the manufacturers' instructions. Expression levels were normalized to the average of U6-snuRNA. MiR-27a levels were calculated as fold change (2^−ΔΔCT^) with respect to normal controls. The mean value of miR-27a expression in glucose-free cultured cells was used as the calibrator. Target-specific reverse transcription and Taqman microRNA assays were performed using the Hairpin-it^TM^ miRNA qPCR Quantitation Kit (GenePharma, Suzhou, China) according to the protocol. The reactions were performed using the ABI PRISM 7500 FAST Real-TIME PCR System (Applied Biosystems, Carlsbad, CA, USA) with results normalized to U6-snuRNA expression. The 2^−ΔΔCt^ method was used to calculate the relative expression. All experiments were performed in triplicate.

### Western blot analysis

Lysates from the cells and microdissected renal tubules from each experimental group were separated in parallel on two 10% denaturing sodium dodecyl sulfate-polyacrylamide gels, transferred onto nitrocellulose membranes, blocked with 5% nonfat milk in 0.1% tris buffered saline with Tween-20 (TBST), and probed using antibodies at 4°C overnight. Primary antibodies against PPARγ (1:100, ab19481), TGF-β1 (1:100, ab27969), total Smad3 (1:100, ab40854), Smad3 (phospho S213) (1:100, ab63403), CTGF (1:100, ab6992), Fibronectin (1:200, ab2413), Collagen I (1:200, ab6308) and beta Actin (1:200, ab6276) were purchased from Abcam (Cambridge, USA). After extensive washing in TBST buffer, the secondary antibody (horseradish peroxidase-labeled IgG anti-rabbit/mouse antibody, Invitrogen, Cambridge, MA) was used at 1:3000 dilution for 1 hour at room temperature. The supersignal-enhanced chemoluminescent substrate (Pierce Biotechnology, Inc., Rockford, IL) was applied to the probed membrane and exposed for 10 minutes before the protein bands were visualized on radiograph films (Super Rx, Fuji Photo Film, Tokyo). Quantification was performed by measurement of the intensity of the bands using ImageJ analysis software (National Institutes of Health, Bethesda, MD).

### Patients and renal biopsy studies

Total seventy-two renal biopsy samples were obtained from type 2 diabetic patients including 15 from the Division of Nephrology in Zhujiang Hospital of Southern Medical University in Guangzhou and 57 from the Division of Nephrology in the First Affiliated Hospital of Inner Mongolia Medical University in Hohhot from 2010 to 2015. The inclusion criteria were: 1) type 2 diabetic patients with no history of using renal toxic or herbal medicine; 2) the indications for performing the renal biopsy were proteinuria with or without microscopic hematuria and fast drop in renal function; 3) diabetic patients with no complications of other renal diseases. The Ethnics Committee from Southern Medical University and Inner Mongolia Medical University specifically approved the use of patient tissue samples in this study and written informed consent was obtained from each patient.

In all specimens, the morphological diagnosis of DN was confirmed by two individual renal pathologists (JG and XB). Normal human renal tissues (*n* = 5) from distant portions of kidney tumor were used as controls. Serum samples from the 30 DN patients and 30 healthy volunteers were collected. Biological parameters including blood glucose level, BUN, serum creatinine, 24 hour proteinuria, urinary NAG and eGFR were analyzed.

### Immunofluorescence and immunohistochemical analysis

NRK52E cells, tissue samples from the patients and rats were labeled with antibodies to PPARγ (1:100), TGF-β1 (1:100), phospho-Smad3 (1:100), CTGF (1:100), Fibronectin (1:100) and Collagen I (1:100). For immunofluorescence staining, Alexa Fluor 594-conjugated goat anti-mouse IgG and Alexa Fluor 488-conjugated goat anti-rabbit IgG (1:1000, Invitrogen, Cambridge, MA) were used for secondary antibodies, nuclei were counterstained with 4,6-diamidino-2-phenylindole (DAPI, Sigma-Aldrich, St. Louis., MO) and coverslipped with aqueous mounting medium (CTS011, BD Bioscience, Minneapolis, MN). For immunohistochemistry, EnVision™ Detection Systems Peroxidase/diaminobenzidine (DAB), Rabbit/Mouse kit (K4065, Dako, Carpinteria, CA) was used. Nuclei were counterstained with hematoxylin and coverslipped with Permount mounting medium (00–4960–56, eBioscience, San Diego, CA).

Samples were evaluated semiquantitatively by systematically selecting without bias twenty fields for analysis. Images were taken with a BX51 light microscope (Olympus, Tokyo) with appropriate filters. Staining intensity was measured using Image J analysis software (Image J 1.44, National Institute of Health). PBS instead of primary antibodies served as a negative control.

### Evaluation of renal tubulointerstitial fibrosis (TIF)

Five-μm thick paraffin sections were cut for Masson's trichrome stain (MTS). Area of TIF was measured using the Image J analysis software (Image J 1.44, National Institute of Health) by evaluating areas of the injured tubules and interstitium and recorded as interstitial injury score.

### Statistical analysis

Data are presented as mean ± SD. Independent–Samples *T* Test and One-Way ANOVA were used to test statistical significance between groups. Pearson correlation analysis was used to analyze correlations between plasma miR-27a and biological parameters. All statistical tests were performed using SPSS 12.0 (SPSS, Inc., Chicago, IL, USA). The significance level is set at 0.05 to indicate statistical significance.
